# Effect of vinyl silane modification on thermal and mechanical properties of starch-polyvinyl alcohol blend

**DOI:** 10.1080/15685551.2019.1678223

**Published:** 2019-10-17

**Authors:** Ravindra V Gadhave, Prakash A. Mahanwar, Pradeep T. Gadekar

**Affiliations:** Department of Polymer and Surface Engineering, Institute of Chemical Technology, Mumbai, India

**Keywords:** Starch, polyvinyl alcohol, citric acid, vinyltrimethoxysilane, polymerization

## Abstract

This study aims to observe the effect of addition of silane coupling agent on polyvinyl alcohol and starch-PVA blend. Starch and PVA blend with citric acid addition was prepared. Silane-modified polymer was obtained by treating polyvinyl alcohol and starch-PVA blend with *Trimethoxyvinylsilane*. The blend has been tested against the canarium wood substrate for tensile strength. A further property like viscosity has also been evaluated. Analytical tests such as DSC and DMA proved the phenomenon of cross-linking, having shown an increase in glass transition temperature and area under the curve of tan delta. The efficient and novel method for polymerization of vinyl groups present in the PVA and PVA-starch blends has contributed to better adhesion on the wood substrate and also better cohesion between the chains.

## 1. Introduction

Polyvinyl alcohol (PVA) has been known to possess a high degree of orientation and consequently high crystallinity in its structure [1–5]. Over the years PVA has been in the industrial use for various paper, textile and adhesive industries [6,7]. Another polymer which is highly similar to PVA in structure and naturally sourced with minimum cost for processing is starch [8].

Starch (S) is known to exist abundantly in nature and is readily available in nature. It has many advantages such as low cost, renewable, abundant supply and environmental easily available. It is widely used in food, paper-making, fine chemicals, packing material industry, adhesives, etc. [9]. It is one of the most abundant biomass materials in nature [10]. Chemically modified starches extend the range of physical properties available for various uses because they exhibit excellent physicochemical properties that are markedly altered from those of their parent starches. However, starch bonding capacity is insufficient for gluing wood. Nevertheless, these starch-based wood adhesives still lack the high bonding strength and water resistance.

The bonding strength and thermal properties of the adhesive have improved significantly confirming the positive effect of adding silane coupling agent and olefin monomer to the adhesive system [11]. Silane coupling agent is commonly used to strengthen the interfacial interaction between starch hydroxyl groups [12,13]. For the adhesive, organo-silanes can combine with starch to form a very thin coat, and the C-Si-O- connects with the hydrogen bonding in the surface of wood [14–16].

**Figure 1.**
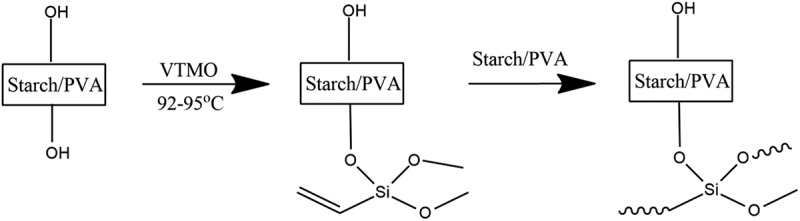
Cross-linking mechanism of Starch/PVA with VTMO.

The alkoxysilanes have been demonstrated to be able to directly react with – Si-OH groups of silica thereby forming – Si-O-Si- bonds without any requirement of pre-hydrolysis [17]. However, silanes do not undergo the same reaction with the hydroxyl groups of starch/PVA even at high temperature. This has been attributed to lower acidity of starch/PVA hydroxyl groups compared with silanol. In addition, starch/PVA is generally un-reactive to many chemicals and the OH groups have very low accessibility [18]. Based on the fact, an optional strategy is to activate the alkoxysilane by hydrolyzing the alkoxy groups thereby forming more reactive silanol groups [19]. As a result, the silanol may react with the hydroxyl groups of fibers or condense themselves on the surfaces of fibers and/or in the cell walls forming macromolecular network as shown in Figure 1 [20].

The purpose of the study was to investigate the bonding strength and thermal properties of silane-modified polymer obtained by treating polyvinyl alcohol and starch-PVA blends with Trimethoxyvinylsilane.

## 2. Experimental

### 2.1 Materials

PVA (Degree of hydrolysis of 86.5% to 89%) was obtained from Kuraray Co., Ltd India. Normal corn starch [moisture content, 10–12%; amylose content, 25–30% (w/w, dry basis); purity, >99%] was obtained from Sanstar Bio-polymer Ltd. These raw materials were kept in dry environment to avoid absorption of moisture from environment. Trimethoxyvinylsilane [VTMO] was obtained from Evonik, India.

### 2.2 Preparation method

Corn starch: PVA blends (S: PVA) were made as shown in Table 1 at temperature 92–95°C for about 2.5 h with continuous stirring with overhead stirrer. Dissolution in water was done in presence of citric acid. The solution was stirred at this temperature for 2 h and then the temperature was reduced to 80°C and 15 g of VTMO was added into the solution. After homogenizing for 15 min, the reaction was initiated by the addition of 10 g of 3% aqueous potassium peroxodisulphate solution. The temperature was kept at 80°C for 4 h and then the mixture was cooled to 25° C. A clear polymer solution was obtained. Composition is shown in Table 1.

**Table 1.** Composition of SM series with and without addition VTMO.

Ingredients	PVA	SM PVA	SPVA	SM SPVA
Water	88	88	88	88
Starch	0	0	10	10
PVA	18	18	8	8
Potassium disulfate (3% solution)	0	5	0	5
Citric Acid	0	0	0	0.5
VTMO	0	1.5	0	1.5

## 3. Characterization and testing

### 3.1. Brookfield viscosity

A Brookfield DV1 Viscometer was used for calculating the viscosities.

### 3.2. Dynamic mechanical analysis (DMA)

DMA was performed using DMA Q800. A thin film of 200 micron was first prepared by applying it on a PTFE sheet. The film was kept for curing at room temperature for 24 h. After curing, the film was peeled off from the surface and kept on the DMA sample holder. It is a technique used to study and characterize materials. It is most useful for studying the viscoelastic behavior of polymers. A sinusoidal stress was applied and the strain in the material was measured in terms of complex modulus. The temperature of the sample or the frequency of the stress are often varied, leading to variations in the modulus. This approach can be used to locate the glass transition temperature of the material as well as to identify transitions corresponding to crosslinking between the molecules.

### 3.3. Differential scanning calorimetry (DSC)

A Perkin Elmer instrument Q100 DSC was used for estimating the Tg of the polymer.

### 3.4. Tensile shear strength

Tensile strength was checked with the help of UTM Tinus Olsen H25KT. A constant amount of adhesive was applied on 20 mm × 10 mm area of one end of the steamed beech wood pieces ensuring that it properly wets the surface. The adhesive coated steamed beech wood pieces were assembled in such a way that the grains of two pieces were oriented in parallel. Each end of the sample was held by vice grips and pulled apart at a controlled rate and tensile strength for the adhesive bond was recorded.

## 4. Results and discussion

### 4.1. Viscosity (RT)

**Figure 2.**
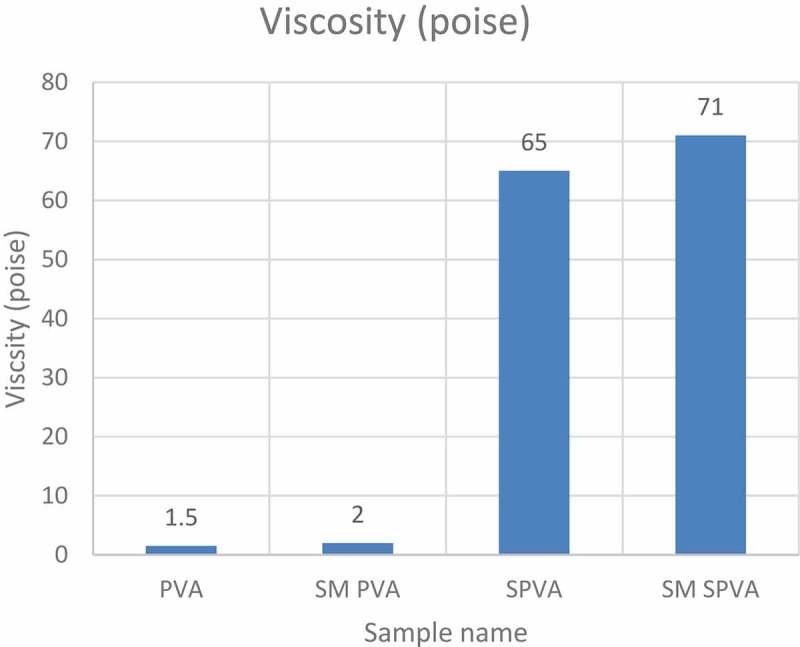
Viscosity at increasing concentration of vinyl silane.

The cross-linking reaction between Starch-PVA with VTMO silane caused an increase in the viscosity of the glue as shown in Figure 2.

The presence of citric acid in the starch PVA blend, cross-linking of hydroxyl groups and polymerization of vinyl group in silane has increased the viscosity (**SM SPVA**). This is seen because citric acid helps in gelatinization of starch molecules, thereby helping the vinyl silane to create a networked structure.

The viscosity for unmodified PVA solution (PVA) is comparable to the PVA solution modified with vinyl silane (SM PVA) as shown in Table 2.

**Table 2.** Viscosity data of various samples.

Sample name	Viscosity (poise)
PVA	1.5
SM PVA	2.0
SPVA	65
SM SPVA	71

### 4.2. Dynamic mechanical analysis

**Figure 3.**
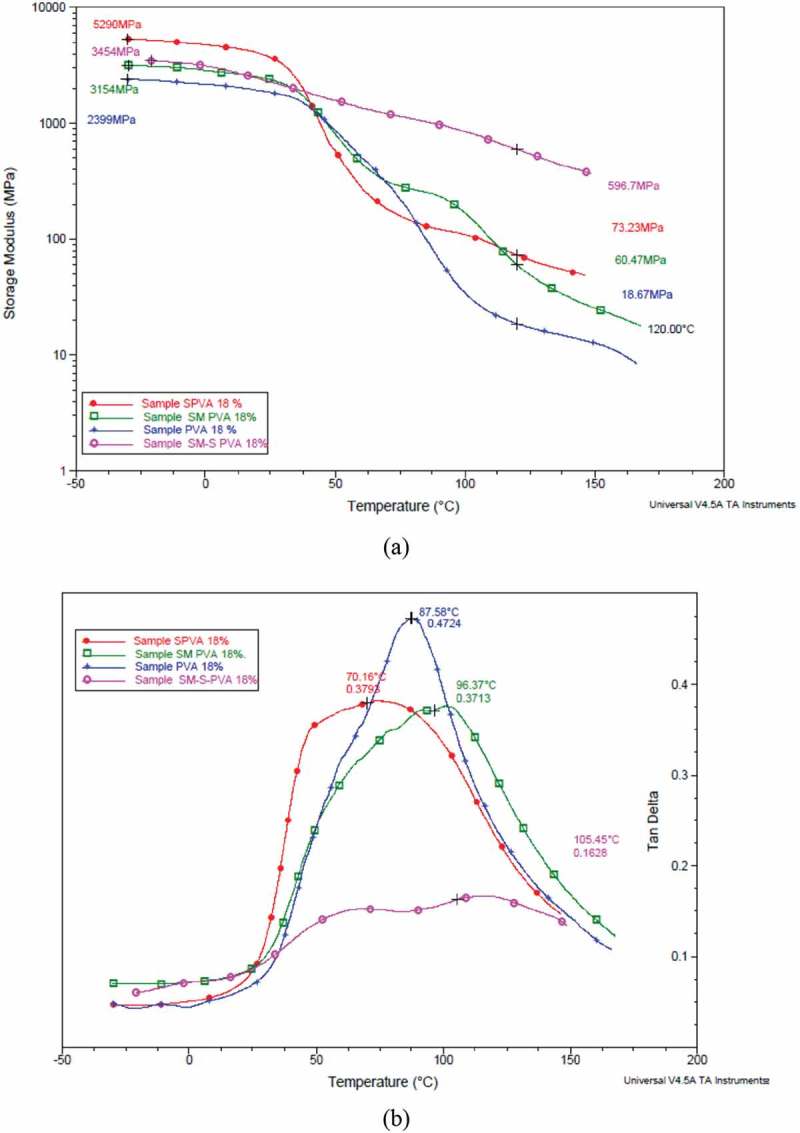
(a) Overlaid thermogram of storage modulus (E’) of samples (b) Overlaid thermogram of tan delta of samples.

All four samples showed transition at 90°C corresponding to polyvinyl alcohol. The storage modulus curve shows the highest value for SM PVA throughout the glassy and rubbery region and this confirms the cross-linking effect on the hydroxyl groups of starch and PVA (Figure 3(a)).

The crosslinking has also been shown by the tan delta curve where there is an evident shift in the peak of the curve towards the higher temperature (from 87.58°C to 96.37°C and 70.16°C to 105.45°C). This shift is a representation of the transition temperature change which has caused due to the cross-linking of silane (Figure 3(b)).

### 4.3. Differential scanning calorimetry

The crosslinking and polymerization reaction of S-PVA with vinyl silane has caused an increase in the glass transition temperature of the solution as shown in Table 3.

**Table 3.** Glass transition temperature data of various samples.

Sample name	Tg
PVA	68.25
SM PVA	74.25
SPVA	72.00
SM SPVA	82.73

**Figure 4.**
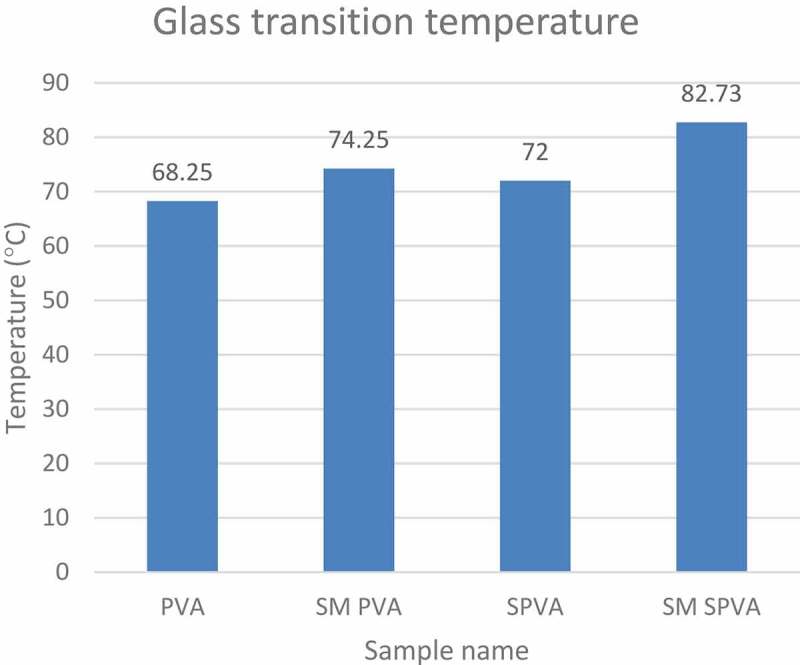
Glass transition temperature at increasing concentration of vinyl silane.

While comparing the glass transition temperature, it was observed that the Tg for SM PVA was greater than PVA. In Starch PVA blend, Tg for SM SPVA was greater than SPVA. From this the role of vinyl silane as a cross-linker and self polymerization additive for starch & PVA is evident as shown in Figure 4.

### 4.4. *Tensile shear strength (can to can*)

**Figure 5.**
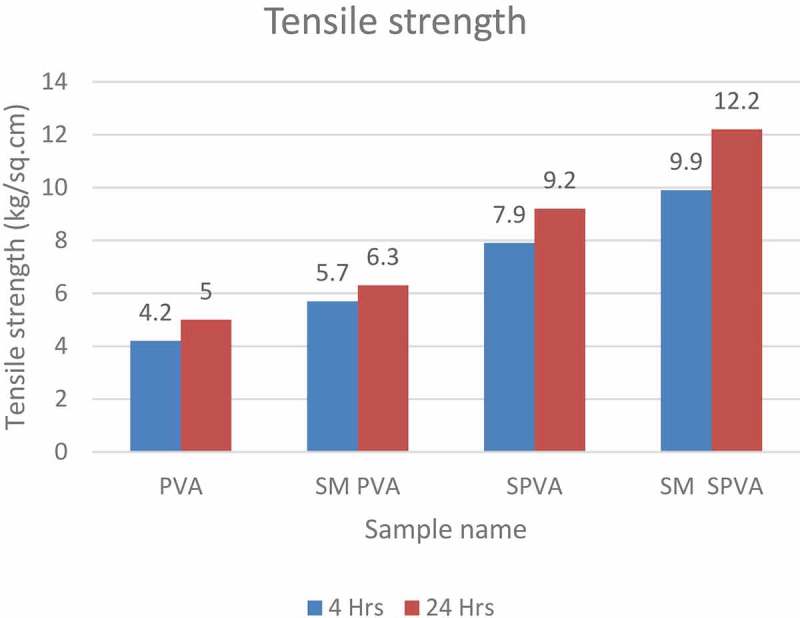
Tensile strength at increasing concentration of vinyl silane.

The crosslinking of S-PVA with vinyl silane caused an increase in the tensile strength of the composition as shown in Figure 5.

Among all the samples, PVA has the least tensile strength. The control sample SPVA having no silane groups shows less tensile strength compared to SM SPVA. As seen from Figure 5, the highest tensile strength is observed for SM SPVA having silane modification (Table 4). This is due to the crosslinking and self polymerization of vinyl silane.

**Table 4.** Tensile shear strength data of various samples.

Sample name	4 Hrs	24 Hrs
PVA	4.2	5.0
SM PVA	5.7	6.3
SPVA	7.9	9.2
SM SPVA	9.9	12.2

## 5. Summary and conclusion

The reaction of vinyl silane with S-PVA and polymerization of vinyl group has led to increase in the thermal and mechanical properties. The viscosity showed major increase in SM SPVA due to the presence of vinyl silane. Tensile strength has shown a considerable increase in the properties of the blends due to better adhesion to the wood substrates. An increase in area under the tan delta curve combined with greater storage modulus at 110°C confirms the cross-linking mechanism. Also, the values of Tg have been shifted towards the higher temperature due to the cross-linking. Thus, the vinyl silane modification of S-PVA can be a promising tool to increase the performance properties of blend without losing its original properties.
